# Supplementation with *Astragalus* Root Powder Promotes Rumen Microbiota Density and Metabolome Interactions in Lambs

**DOI:** 10.3390/ani14050788

**Published:** 2024-03-02

**Authors:** Pengyang Shao, Yuzhu Sha, Xiu Liu, Yanyu He, Fanxiong Wang, Jiang Hu, Jiqing Wang, Shaobin Li, Xiaowei Chen, Wenxin Yang, Qianling Chen, Min Gao

**Affiliations:** 1 Gansu Key Laboratory of Herbivorous Animal Biotechnology, College of Animal Science and Technology, Gansu Agricultural University, Lanzhou 730070, China; shaopengyang666@163.com (P.S.); shayz@st.gsau.edu.cn (Y.S.); m19893318751@163.com (F.W.); huj@gsau.edu.cn (J.H.); wangjq@gsau.edu.cn (J.W.); lisb@gsau.edu.cn (S.L.); cxw20002022@163.com (X.C.); aaaaa0108@163.com (W.Y.); chenqianling223@163.com (Q.C.); gm12017101@163.com (M.G.); 2 School of Fundamental Sciences, Massey University, Palmerston North 4410, New Zealand; y.h@massey.ac.nz

**Keywords:** *Astragalus* root powder, rumen microbiota, metabolome, lambs

## Abstract

**Simple Summary:**

The gut microbiota plays an important role in animals. Metabolomics analysis was used in this study to investigate the rumen microbiota density and metabolome interactions in lambs supplemented with *Astragalus* root powder; the density of the rumen microbiota and its relationship with the metabolome in lambs supplemented with *Astragalus* root powder were evaluated. The results showed a significant correlation between the rumen microbiota and its metabolome in lambs. These findings have important implications for livestock nutrition and management practices, particularly in terms of improving overall productivity and profitability.

**Abstract:**

The gut microbiota is highly symbiotic with the host, and the microbiota and its metabolites are essential for regulating host health and physiological functions. *Astragalus*, as a feed additive, can improve animal immunity. However, the effects of *Astragalus* root powder on the rumen microbiota and their metabolites in lambs are not apparent. In this study, thirty healthy Hu sheep lambs with similar body weights (17.42 ± 2.02 kg) were randomly selected for the feeding experiment. Lambs were fed diets supplemented with 0.3% *Astragalus* root powder, and the rumen microbiota density and metabolome were measured to determine the effects of *Astragalus* on the health of lambs in the rumen. The results showed that the relative abundance of *Butyrivibrio fibrisolvens* (*Bf*), *Ruminococcus flavefaciens* (*Rf*), *Succiniclasticum* (*Su*), and *Prevotella* (*Pr*) in the rumen was increased in the *Astragalus* group (*p* < 0.01), and metabolic profiling showed that the metabolites, such as L-lyrosine and L-leucine, were upregulated in the *Astragalus* group (*p* < 0.01). KEGG functional annotation revealed that upregulated metabolites were mainly enriched in the pathways of amino acid metabolism, lipid metabolism, fatty acid biosynthesis, and bile secretion in the *Astragalus* group, and downregulated metabolites were enriched in the pathways of methane metabolism and other pathways. Correlation analysis revealed that butyric acid was positively correlated with *Roseburia* and *Blautia* (*p* < 0.05) and negatively correlated with *Desulfovibrio* (*p* < 0.05). Thus, by analyzing the interactions of *Astragalus* root powder with the density of rumen microorganisms and their metabolites in lambs, it was shown that *Astragalus* root powder could improve the structure of rumen microbiota and their metabolites and then participate in the regulation of amino acid metabolism, lipid metabolism, immune metabolism, and other pathways to improve the efficiency of energy absorption of the lambs.

## 1. Introduction

Lamb is characterized by high protein content, low fat content, and low cholesterol content and is one of the primary animal protein sources in humans [1]. Along with the rapid development of the sheep meat industry, early weaning technology has become a key technology for improving the efficiency and profitability of the intensive sheep-rearing industry [2]. However, lambs are subjected to physiological and environmental influences during weaning, adversely affecting their growth, development, and health, resulting in a stress response [3]. Stress decreases the immunity of lambs, resulting in slow growth, diarrhea, and increased mortality [4] and causing substantial economic losses to the sheep industry. The addition of antibiotics can alleviate this phenomenon, but the excessive addition of antibiotics can lead to drug residues in livestock products, which can cause serious harm to human health [5]. The Chinese government’s policy of banning antibiotics has led to the introduction of new policies. The introduction of the Chinese government’s anti-antibiotic ban policy has made the substitution of antimicrobials a hot topic in the animal feed industry. Therefore, finding a natural and harmless feed additive that can help lambs survive weaning stress while improving their intestinal health and performance and realizing greater economic benefits is a better choice for replacing antibiotics in the context of antibiotic bans. Chinese herbs are rich in resources and are potential alternative products in the feed industry. Xu et al. [6] reported that the addition of a herbal dietary supplement (containing paeonols, liquorice, dandelion, and tea polyphenols) to feed improved the growth performance, immunity, and antioxidant capacity of piglets, as well as the composition of the intestinal microbiota. *Astragalus* roots mainly contain saponins, flavonoids, polysaccharides, alkaloids, β-sitosterol, amino acids, anthraquinones, iron, calcium, phosphorus, selenium, zinc, copper, manganese, and other trace elements [7], These roots also have antioxidant, immunomodulatory, and cardiovascular disease relief effects, and the addition of *Astragalus* to feeds can improve animal performance, antioxidant capacity, and immunity, improve the intestinal microbiota, and relieve stress [8,9,10].

The rumen, the hallmark organ of ruminants, plays a vital role in nutrient absorption, immunity, and host metabolism [11]. Moreover, a large number of microbes, including bacteria, archaea, fungi, and protozoa, are present in the rumen. These bacteria participate in the catabolism of nutrients such as proteins, starch, and cellulose and provide volatile fatty acids (VFAs), microbial proteins, and other metabolites to the host [12,13,14]. In addition to providing essential nutrients to the host, these metabolites regulate essential functions such as immunity and energy metabolism [15], which ultimately affects animal performance. Newbold et al. [16] suggested that nutritional intervention in early ruminants can change the structure and function of their microbiota. A previous study showed that adding 0.3% *Astragalus* to the diet improved the rumen microbiota structure of early-weaned lambs, enhanced immunity, and helped lambs survive the stressful phase [17]. However, the mechanisms by which *Astragalus* root powder interacts with the rumen microbiota and metabolome of lambs are not fully understood. Therefore, this study was conducted to determine the density of the rumen microbiota and its metabolome in lambs. The effect of *Astragalus* root powder on the rumen metabolome of lambs and the interaction between the rumen microbiota and metabolome of lambs were also investigated in conjunction with previous studies.

## 2. Materials and Methods

### 2.1. Test Animals and Materials

The *Astragalus* root was selected as the experimental material in this study. The *Astragalus* root used in this experiment was produced from the ecological planting base of Runfengyuan Agriculture and Animal Husbandry in Minle Township, Yongdeng County, Gansu Province, China (Altitude: 2650 m, 38′25′ N, 100′48′ E). The *Astragalus* root was crushed by a pulverizer, passed through a 200-mesh sieve, bagged, and sealed. According to the feeding characteristics of fattening sheep, it was made into a granular herbal additive. *Astragalus* root mainly contains saponin, flavonoids, polysaccharides, alkaloids, β-sitosterol, amino acids, anthraquinones, iron, calcium, phosphorus, selenium, zinc, copper, manganese, and other trace elements, and the main biologically active ingredients of *Astragalus* conform to the Pharmacopoeia of the People’s Republic of China [17].

The feeding experiment was carried out in Aonong Pasture, Gulang County, Wuwei city, Gansu Province. Thirty healthy, 45-day-old Hu sheep lambs with similar body weights (17.42 ± 2.02 kg) were randomly selected and divided into two groups. One group was fed a basal diet (control group) and the other group was fed a basal diet supplemented with 0.3% *Astragalus* (*Astragalus* group). The sheep pens were cleaned and disinfected before the start of the experiment, and the fattened sheep in each experimental group were numbered. The test groups were fed individually (15 lambs/pen); area of pen: 400 × 500 cm. A leaky floor was used for the pen floor. The pen was kept clean and hygienic, and the sheep were immunized and dewormed regularly. The test sheep were fed twice a day (06:30 and 18:30) and watered freely. The pretest period was 7 days, and the formal test period was 60 days. The composition and nutrient levels of the basal diet are shown in Table 1 [17].

### 2.2. Sample Collection

At the end of the experiment, 6 of the 15 test lambs in each group were randomly selected. Then, rumen fluid was collected from all 6 lambs. Lambs were fasted and deprived of water for 12 h prior to sample collection. Rumen fluid was collected using a sheep gastric tube rumen sampler, and approximately 5 mL of rumen fluid was collected from each individual. The samples were quickly frozen in a liquid nitrogen tank. The samples were returned to the laboratory and stored at −80 °C.

### 2.3. Rumen Microbiota Density Measurement

The collected rumen fluid samples were thawed at room temperature and mixed. The total rumen microbial DNA of the lambs was extracted using the TIANamp Stool DNA Kit (DP-328). DNA integrity and purity were detected using 1% agarose gel electrophoresis, and OD260/280 values were measured via an ultramicro-spectrophotometer (Therm Nano Drop-2000). *Butyrivibrio fibrisolvens* (*Bf*), *Ruminococcus flavefaciens* (*Rf*), *Fibrobacter* (*Fi*), *Prevotella* (*Pr*), *Succiniclasticum* (*Su*), *Clostridium butyricum* (*Cb*), *Selenomonas ruminantium* (*Sr*), and *Treponema bryanti* (*su*) were selected for quantitative real-time PCR studies using rumen microbial DNA as a template. NCBI BLAST (www.ncbi.nih.gov/BLAST, accessed on 10 March 2023) was utilized to search for bacterial 16S rRNA primer sequences, and Primer 5.0 software was used to design specific primers (Table 2). Bacteria were used as an internal reference, and the bacterial primers used were described by Muyzer et al. [20].

### 2.4. LS-MS/MS Metabolic Profile Determination

The microbial metabolite composition in the rumen of lambs was examined through the use of a liquid chromatography–mass spectrometry (LC-MS) platform. To retrieve the metabolites, 100 μL of the samples was thawed at room temperature and then extracted with 500 μL of an extraction solution containing an internal standard at a concentration of 2 mg/L, using a 1:1 volume ratio of methanol to acetonitrile. After vortexing the extracts for 30 s, the samples were sonicated in an ice-water bath for 10 min and then left to stand at −20 °C for approximately one hour. The samples were then centrifuged at 4 °C for 15 min (7727× *g*), 500 μL of supernatant was removed from the EP tube, and the extract was dried in a vacuum concentrator. The dried metabolite was redissolved in 150 μL of acetonitrile–water solution (1:1) and vortexed for another 30 s. The samples were then sonicated in an ice-water bath again, and the supernatant was removed by centrifugation at 4 °C for 15 min (7727× *g*). Finally, 120 μL of the supernatant was placed into a 2 mL injection bottle, and 10 μL of each sample was mixed into a QC sample for testing. In the course of the metabolomics analysis, a Waters Acquity I-Class PLUS Ultra-High-Performance Liquid (Waters, Milford, CT, USA), in tandem with a Waters Xevo G2-XS QToF High-Resolution Mass Spectrometer (Waters, Milford, CT, USA), was used as the LC/MS system. Additionally, a Waters Acquity UPLC HSS T3 (Waters, Milford, CT, USA) column (1.8 µm 2.1 × 100 mm) was used. The samples were eluted using positive-ion-mode (ESI+) and negative-ion-mode (ESI−) mobile phases consisting of water and 5% acetonitrile, 0.1% formic acid as solvent A, and acetonitrile and 0.1% formic acid as solvent B, at a flow rate of 400 μL/min at 0.35 mL/min. The elution gradient of the subsequent mobile phase (A:B) was adjusted as follows: 0–0.25 min 98–2%, 10.0–13.0 min 2–98%, and 13.1–15.0 min 98–2%. The ion source temperature was set to 150 °C, while the desolvation temperature was set to 500 °C. The flow rates of blowback and desolventization gases were set to 50 and 800 L/h, respectively. To process the raw data collected using MassLynx V4.2, Progenesis QI (version 4.0) software was utilized for operations such as peak extraction and peak pairing. Identification of the compounds was conducted based on the online METLIN database of Progenesis QI (version 4.0) software and Biomark’s self-constructed libraries. The theoretical fragment identifications and mass deviations were maintained within 100 ppm. Principal component analysis and Spearman correlation analysis were employed to assess the reproducibility of the within-group and mass control samples. Classification and pathway information of the identified compounds were obtained by searching the KEGG database. The multiplicity of differences was calculated and compared based on the grouping information, followed by the use of a *t* test to determine the significance of differences for each compound. The R package ropls was utilized to perform OPLS-DA modeling, and the reliability of the model was verified through 200 permutation tests. The VIP values of the models were calculated using multiple cross-validation. Differentially abundant metabolites were screened using a combination of multiple differences, *p*-values, and VIP values from the OPLS-DA model. The screening criteria included FC > 1, *p* < 0.05, and VIP > 1. A hypergeometric distribution test was employed to assess the significance of differentially enriched metabolites based on KEGG pathway enrichment.

### 2.5. Data Analysis

According to the methods of McHardy et al. [21], for the joint analysis of the microbiome and metabolome, PCoA was chosen to downrank the microbiome (genus level) and metabolome. The distance matrices were first calculated using the microbial quantification matrix and the metabolite quantification matrix, respectively, where the distance algorithm for the microbiome was Bayesian distance and that for the metabolite group was the Euclidean distance, and the PCoA was utilized to rank the distances. The coordinates of the characteristic axes in the PCoA results of the microbiome and metabolome were extracted. Procrustes analysis was performed to compare the similarities and variations between the microbiome and metabolome. The metabolic data were downscaled by weighted gene co-expression network analysis (WGCNA), which divides metabolites into different metabolite clusters, and the expression of metabolite clusters is expressed as the median content within the same cluster. In the Pearson correlation analysis with microorganisms, a heatmap was generated, and the results of the correlation analysis were screened for *p* values, which were based on the criterion of CCP < 0.05. The frequency counts of the metabolite clusters/microorganism occurrences were subsequently counted, the correlation results of the metabolite clusters/microorganism with the top 30 frequencies were tabulated, and chord diagrams were plotted. Correlation tests were performed by Spearman correlation analysis using Origin 2022 for fermentation microbiota and VFAs, genus-level rumen microorganisms (top 15), fermentation microbiota, differentially abundant metabolites, and VFAs.

## 3. Results

### 3.1. Rumen Microbiota Density

**Figure 1:**
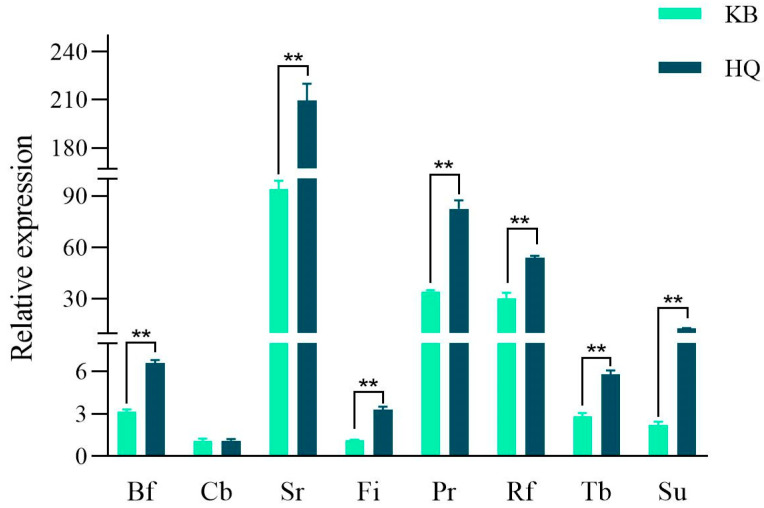
Determination of rumen microbiota density. *Bf*: *Butyrivibrio fibrisolvens*; *Cb*: *Clostridium butyricum*; *Sr*: *Selenomonas ruminantium*; *Fi*: *Fibrobacter*; *Pr*: *Prevotella*; *Rf*: *Ruminococcus flavefaciens*; *Tb*: *Treponema bryanti*; *Su*: *Succiniclasticum*. Note: ** *p* < 0.01.

A total of 14 phylum-level microbiota and 125 genus-level microbiota were found in the *Astragalus* and control groups in a previous study (Data S1) [17]. Quantitative analysis of the screened fermentation-associated microbiota (Figure 1) revealed that the rumen microbiota density was altered by the addition of *Astragalus* to the diets of early weaned lambs. The relative densities of *Butyrivibrio fibrisolvens* (*Bf*), *Selenomonas ruminantium* (*Sr*), *Fibrobacter* (*Fi*), *Prevotella* (*Pr*), *Ruminococcus flavefaciens* (*Rf*), *Treponema bryanti* (*Tb*), and *Succiniclasticum* (*Su*) were significantly (*p* < 0.01) greater than those in the control group. The relative density of *Clostridium butyricum* (*Cb*) was elevated in the *Astragalus* group, but the difference was not significant (*p* > 0.05).

### 3.2. Metabolic Profiling of the Rumen Microbiota

**Figure 2:**
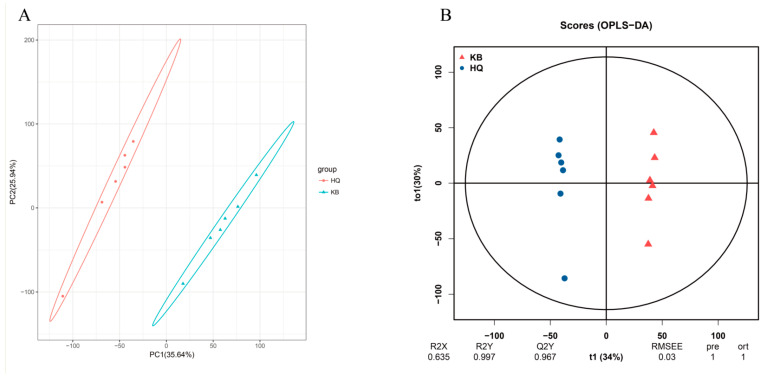
Metabolome data quality control chart of rumen microbiota of lamb. (**A**) PCA; (**B**) OPLS-DA analysis. Note: biological replicates: n = 6.

**Figure 3:**
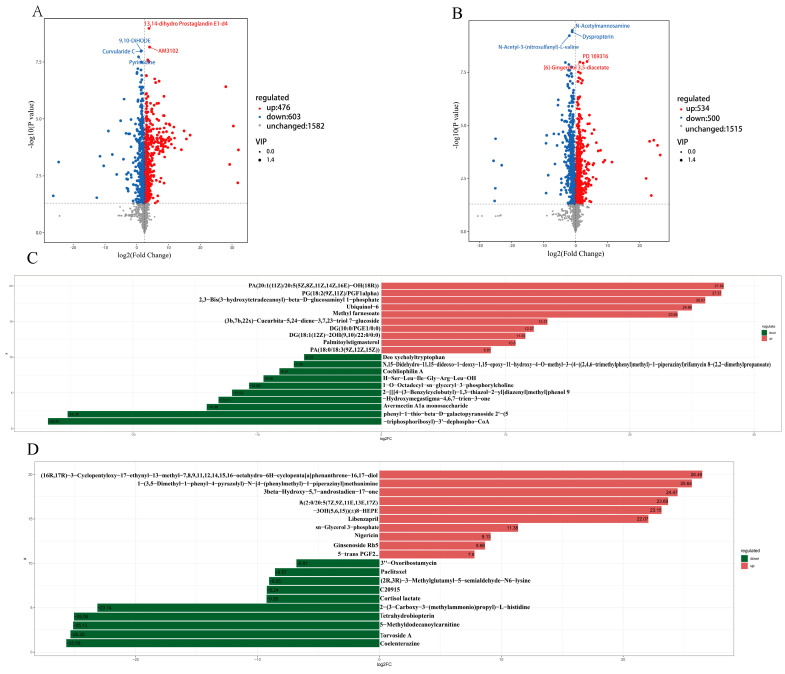
Differential metabolite analysis of lamb rumen microbiota metabolites. (**A**) Volcanic map of positive ion pattern differential metabolites; (**B**) negative map of positive ion pattern differential metabolites; (**C**) column chart of positive ion difference multiples; (**D**) column chart of negative ion difference multiples. Note: biological replicates: n = 6.

A principal component analysis (Figure 2A) revealed that the *Astragalus* root powder affected the in-rumen microbial metabolites in lambs. Orthogonal partial least-squares (OPLS-DA) analysis (Figure 2B) revealed that R2Y and Q2Y were close to 1, which further verified the reliability of the OPLS-DA model for continuing the data analysis. Further differentially abundant metabolite analysis was performed using FC > 1, *p* value < 0.05, and VIP > 1 as screening criteria. A total of 1079 differentially abundant metabolites were identified in the positive ion mode, of which 476 differentially abundant metabolites were upregulated and 603 were downregulated (Figure 3A, Data S2). A total of 1034 differentially abundant metabolites were identified in the negative ion mode, 534 of which were upregulated and 500 were downregulated (Figure 3B, Data S3). Further screening of the top 10 upregulated and downregulated metabolites with the top 10 most common positive and negative ion differences (Figure 3C,D) revealed methyl farnesoate, sn-glycerol 3-phosphate, ubiquinol-6, palmitoylstigmasterol, nigericin, libenzapril, and other upregulated metabolites, and avermectin A1a monosaccharide, 2′-(5-triphosphoribosyl)-3′-dephospho-CoA, paclitaxel, tetrahydrobiopterin, torvoside A, and other downregulated metabolites.

**Figure 4:**
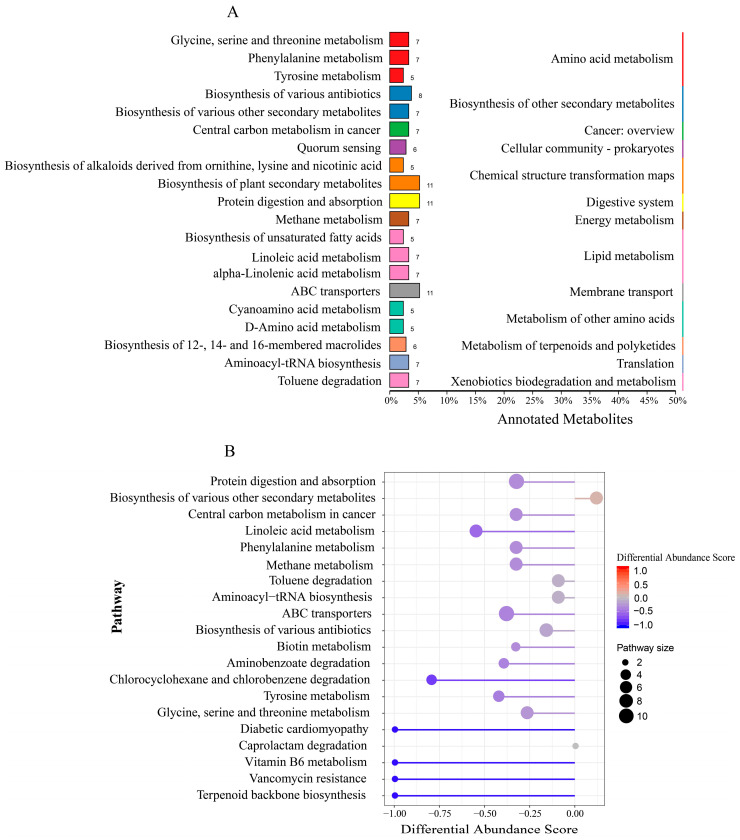
KEGG function analysis of microbial differential metabolites (positive ion mode). (**A**) KEGG annotation of the classification diagram; (**B**) KEGG functional difference abundance score map. Note: biological replicates: n = 6.

**Figure 5:**
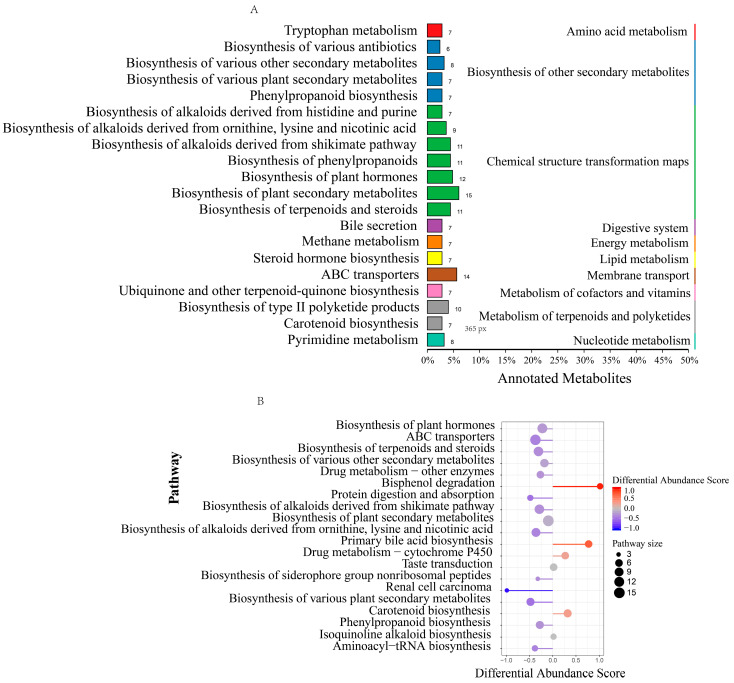
KEGG function analysis of microbial differential metabolites (negative ion mode). (**A**) KEGG annotation of the classification diagram; (**B**) KEGG functional difference abundance score map. Note: biological replicates: n = 6.

In the positive ion mode (Figure 4A), the differentially abundant metabolites were mainly enriched in the pathways of amino acid metabolism, lipid metabolism, the biosynthesis of other secondary metabolites, and energy metabolism. Among them, upregulated differentially abundant metabolites were enriched mainly in the biosynthesis of various other secondary metabolites, and downregulated differentially abundant metabolites were enriched mainly in ABC transporters and protein digestion and absorption (Figure 4B). In the negative ion mode (Figure 5A), the differentially abundant metabolites were enriched in pathways such as the biosynthesis of other secondary metabolites and chemical structure transformation maps. Among them, upregulated differentially abundant metabolites were enriched mainly in bisphenol degradation, primary bile acid biosynthesis, and carotenoid biosynthesis, while downregulated differential metabolites were mainly enriched in ABC transporters and the biosynthesis of plant hormones (Figure 5B).

### 3.3. Rumen Microbiome–Metabolome Interaction Analysis

**Figure 6:**
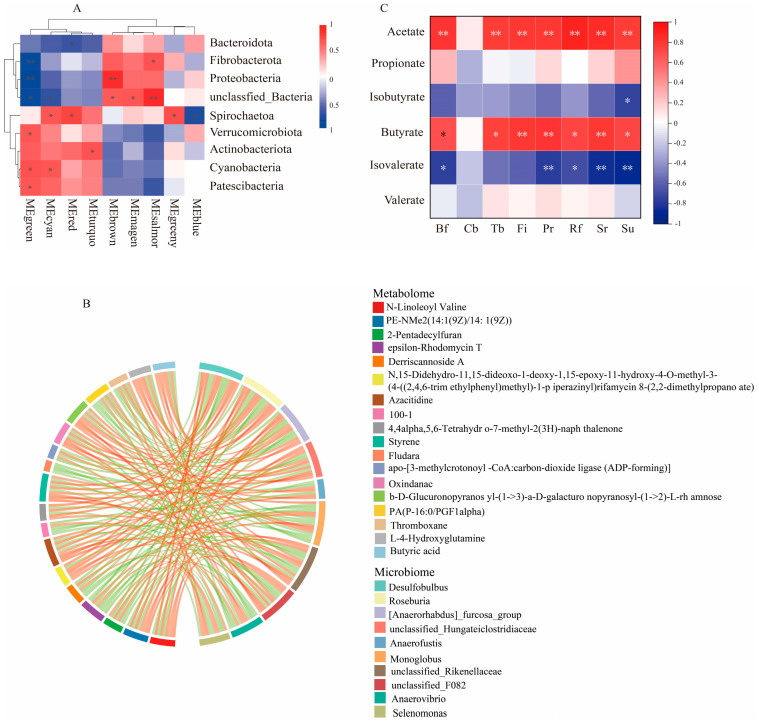
Interaction analysis of rumen microbiome and metabolome. (**A**) Microbe–metabolite correlation heat map; (**B**) differential metabolite/differential microbiota correlation chord diagram; (**C**) heat map of correlation between fermentation microbiota and VFAs. ** means the difference is very significant, * means the difference is significant, no * means the difference is not significant. In the correlation chord diagram, a red string represents a positive correlation and a green string represents a negative correlation.

The rumen microbial metabolome data were subjected to WGCNA dimensionality reduction analysis to classify the metabolites into different metabolite modules, and the eigengene of the corresponding module represented the metabolite module content, which was correlated with the portal-level microbiota (Figure 6A). There was a correlation between the differentially abundant metabolites and genus-level differential microbiota (Figure S1). The data with |CC| > 0.8 and CCP < 0.05 were screened, and correlation chord plots were generated for the top 30 frequencies of differentially abundant metabolites/differential microbiota (Figure 6B). Butyric acid was significantly and positively correlated with *Roseburia* and *Blautia* (*p* < 0.05) and significantly and negatively correlated with *Desulfovibrio* (*p* < 0.05); thromboxane was highly significantly positively correlated with *Succinivibrio*, *Monoglobus*, and the *Lachnospiraceae_XPB1014_group* (*p* < 0.01), and it was highly significantly negatively correlated with *Roseburia* (*p* < 0.01). In conjunction with the findings of a previous study [17], Spearman’s correlation analysis of rumen VFAs and the rumen fermentation microbiota was performed, and the results are shown in Figure 6C. Acetic acid was strongly significantly associated with *Bf*, *Sr*, *Rf*, and *Tb* (*p* < 0.01); butyric acid was significantly positively correlated (*p* < 0.05) with *Bf*, *Rf*, and *Tb*; and *Pr* and *Su* were significantly positively correlated with acetic acid and butyric acid (*p* < 0.05) and highly significantly negatively correlated with isovaleric acid (*p* < 0.01). The above fermentation microbiota was closely related to the production of rumen VFAs.

## 4. Discussion

The rumen is a specialized organ in the digestive system of ruminants that contains a large number of microbiota, such as bacteria, archaea, and fungi [12]. These microorganisms play an essential role in helping the host digest cellulose and other complex carbohydrates [14]. *Bf* are strictly anaerobic Gram-negative bacteria that produce butyric acid, as well as acetic acid and lactic acid, mainly from cellulose as a substrate [22]. *Sr* are strictly anaerobic Gram-negative bacteria that function in the same way as *Bf* [23]. It has been shown that *Sr* can produce propionic acid using substances such as acetic acid and glycerol as substrates [24]. In this study, the *Astragalus* root powder significantly increased the contents of *Bf* and *Sr*, which can promote fermentation of the rumen to produce VFAs such as acetic acid, propionic acid, and butyric acid, which can provide energy for lambs. Moreover, when *Sr* was cocultured with *Rf*, modified cellulose catabolism was accelerated by the conversion of succinic acid, a metabolite of *Rf*, to propionate [25]. *Tb* is a rumen spirochete that interacts with fiber-degrading bacteria. Stanton et al. [26] reported that cocultivating *Tb* with *Su*, *Tb* was able to promote the breakdown of cellulose by *Su*. In turn, *Su* was able to catabolize cellulose to produce succinic acid, which in turn was converted to propionic acid, a precursor of gluconeogenesis [27]. In the present study, both the *Tb* and *Su* content were significantly elevated in the *Astragalus* group, and these two microbiota acted synergistically to accelerate the catabolism of fibrous material in lambs, thereby meeting the energy requirements of the organism. It has been shown that *Pr* has the ability to hydrolyze starch and proteins [28,29] and ferment them to produce acetic acid and succinic acid. Datchary et al. [30] concluded that *Pr* promotes hepatic glycogen storage in the body, and the significant elevation of *Pr* content in the *Astragalus* group favors the energy requirements of lambs and accelerates energy deposition.

A symbiotic solid relationship exists between animal gut microbes and their hosts [31], and the microbiota interacts with a variety of physiological functions in the host through its metabolites [32]. Whereas dietary nutrients significantly influence rumen microbiota structure, a previous study showed that the addition of *Astragalus* improved the rumen microbiota structure [17]. Therefore, in this study, we further analyzed the effect of *Astragalus* root powder on the metabolic function of the rumen microbiota of lambs and revealed that there were some differences in the rumen metabolites of lambs under different ionic modes (positive and negative ions). Tyrosine is an essential ketogenic gluconeogenic amino acid in the body and plays a vital role in protein, lipid, and carbohydrate metabolism [33]. An increase in the tyrosine concentration promotes the synthesis of dopamine (DA) as well as norepinephrine [34] because tyrosine is catalyzed by tyrosine hydroxylase to produce the precursor of DA, dihydroxyphenylalanine, i.e., DOPA. DOPA is catalyzed by DOPA decarboxylase to produce DA, which is, in turn, converted to norepinephrine [35]. An increase in DA, on the other hand, promotes the synthesis and secretion of growth hormone, which in turn promotes animal growth [36]. In the present study, the differentially abundant metabolite L-tyrosine was highly significantly upregulated in the *Astragalus* group (Data S2), which affected the downstream metabolites, and elevated levels of L-tyrosine may have a favorable effect on the growth of lambs. In addition, L-Leucine was upregulated in the *Astragalus* group in this study (Data S2). A large number of studies have demonstrated that Leucine can regulate protein metabolism, oxidative energy supply, and immunomodulation [37,38,39] through its role as a regulator of animal protein synthesis and lipid deposition. Zhang et al. [40] suggested that leucine could be used as a new strategy to improve carcass quality.

The differentially abundant metabolites between *Astragalus* and control groups were found to be enriched mainly in the metabolic pathways of amino acid metabolism, lipid metabolism, and energy metabolism according to KEGG pathway analysis. The results of this study showed that lipid metabolism was active in the rumen, in which upregulated metabolites were mainly enriched in the pathways of biosynthesis of unsaturated fatty acids, linoleic acid metabolism, alpha-linolenic acid metabolism in the positive mode, arachidonic acid metabolism, and fatty acid biosynthesis pathways. In the negative mode, upregulated metabolites were enriched in the glycerolipid metabolism pathway. Among them, linoleic acid and alpha-linolenic acid are mammalian essential fatty acids that must be ingested through the diet [41], and Wang et al. [42] reports that linoleic acid and alpha-linolenic acid synergistically regulate endogenous fatty acid levels and thus maintain lipid homeostasis in mice. A study in tilapia revealed that high alpha-linolenic acid concentrations favored muscle quality, low temperature, and disease resistance [43]. Other lipid metabolic pathways are involved mainly in regulating the antimicrobial and microbial hydrogenation of fatty acids and altering fatty acid uptake [44], suggesting that *Astragalus* root powder promotes lipid metabolism in lambs. Moreover, the present study showed that some upregulated metabolites in the *Astragalus* group were mainly enriched in the amino acid metabolism metabolic pathway, such as arginine and proline metabolism; glycine, serine, and threonine metabolism; and the phenylalanine, tyrosine, and tryptophan biosynthesis pathway. Amino acids in the rumen have been reported to be the main precursors for protein and peptide synthesis, mainly from diets and trace proteins [45]. Among them, proline plays a vital role in protein synthesis, metabolism, antioxidation, and immune responses in the body [46]; arginine, as a semi-essential amino acid, is involved in biological processes such as ammonia detoxification, hormone secretion, and immunomodulation in the body [47]. These differentially abundant metabolites were enriched mainly for functions related to amino acid synthesis and metabolism and lipid metabolism, suggesting that *Astragalus* root powder can promote metabolism, immunity, antioxidant activity, and other functions. In addition, the present study revealed that upregulated metabolites were enriched in the metabolism of xenobiotics by cytochrome P450 and involved bile secretion in the regulation of the immune pathway [48], and bile has been reported to protect organisms from intestinal infections through the secretion of immunoglobulin A (IgA) and inflammatory cytokines and the stimulation of the intrinsic immune system of the intestinal tract [49]. Therefore, *Astragalus* root powder can promote bile secretion and enhance the function of the rumen epithelial immune barrier in lambs. Methane produced by rumen methanogen fermentation accounts for approximately 2–12% of the total energy intake [50], and this loss of energy cannot be used for activities such as the growth and reproduction of the animal. Matthews et al. [51] suggested that reducing methane emissions from ruminants could reduce the impact on the environment and increase the productivity of the animals. In this study, metabolites enriched in the methane metabolism pathway were downregulated in the *Astragalus* group, suggesting that the *Astragalus* root powder was effective at slowing energy loss.

**Figure 7:**
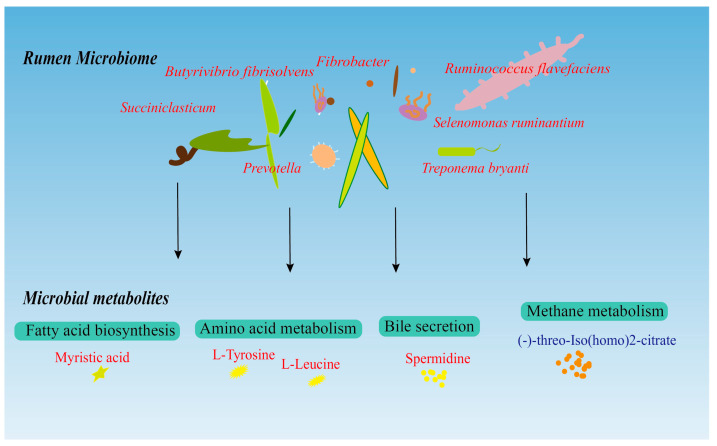
Diagram of the mechanism of rumen microbiota–metabolite interactions. Text in red means upregulated in the *Astragalus* group, the text in blue means downregulated.

Further joint analysis of the microbiome and its metabolome revealed important interactions between them. At the phylum level, the phyla Fibrobacterota, Proteobacteria, and Spirochaetota were correlated with the metabolite module. Fibrobacterota is considered to be an essential bacterial phylum for degrading lignin and cellulose in the rumen of ruminants [52,53] and can help to accelerate the decomposition of fibrous material in the diet of early weaned lambs, resulting in the production of energy metabolites. At the genus level, *Roseburia* species are important butyric acid-producing bacteria [54], and butyric acid, as one of the main VFAs in the rumen, plays an anti-inflammatory role as well as a metabolic regulatory role while providing energy to the organism [55,56,57]. *Blautia* plays an important role in metabolic and inflammatory disorders as well as in biotransformation [58]. In this study, butyric acid was significantly and positively correlated with *Roseburia* and *Blautia*, indicating that *Astragalus* was able to improve the microbiota structure and influence its metabolites, which in turn had an effect on energy deposition and immune function in lambs (Figure 7).

## 5. Conclusions

There were significant differences in rumen microorganisms and metabolites, in which the relative abundance of fiber-degrading bacteria, such as *Bf*, *Rf*, and *Su*, increased in the *Astragalus* group, which promoted the decomposition and metabolism of cellulose materials in the rumen, and provided energy materials for the host. Metabolites such as L-tyrosine and L-leucine were upregulated in the *Astragalus* group. Enrichment analysis of the KEGG pathway showed that *Astragalus* root powder could promote amino acid metabolism, lipid metabolism, fatty acid biosynthesis, and bile secretion, and reduce methane metabolism in lambs, thus playing an important role in metabolism, physiological regulation, and immunity in lambs. *Astragalus* root powder is able to improve the structure of the rumen microbiota and promote its interaction with the metabolome, which accelerates the efficiency of nutrient uptake and the acquisition of energy by the host. This change could positively affect the health of lambs, and further new ideas for antibiotic-free farming in lambs.

## Figures and Tables

**Table 1:** Basic diet composition and nutrient levels (as-fed basis, %).

Ingredients	Content
Corn	40.00
Spray corn husks	10.00
Rice alcohol grain	10.00
A powder	10.00
Soybean meal	8.50
Corn germ meal	8.00
Rapeseed dregs	3.00
Cotton pulp	6.00
Limestone	2.30
Calcium bicarbonate	0.80
Premix ^1^	1.40
Total	100.00
Nutrient levels ^2^	
DE ^3^, MJ/kg	12.95
Crude protein ^4^	≥18.00
Crude ash ^4^	≤12.00
Ca	0.60~1.30
P	≥0.50
NaCl	0.50~1.50
Lysine	≥0.60

^1^ The premix provides the following diet per kg: feed-grade sodium chloride, 20.00 mg; with elder brother, 2.00 mg; ruminant multidimensional FV651, 0.80 mg; antioxidants, 0.60 mg; lysine, 2.20 mg; in ammonia, 0.60 mg; composite spore, 0.60 mg; special enzyme for ruminant T720, 0.40 mg; clostridium butyric acid, 0.20 mg; mold inhibitor, 0.60 mg. ^2^ Nutrient levels are calculated values. ^3^ DE = digestive energy [18], calculated from China Feed Database data on the composition of the base ration ingredients. ^4^ Crude protein (method 984.13) and crude ash content (method 942.05) were measured according to Association of Official Analytical Chemists [19]).

**Table 2:** Primer sequences of microorganisms.

Gene	Primer (5′-3′)	Length	Annealing Temperature	Login ID
*Bacterium*	F: CCTACGGGAGGCAGCAG	181 bp	60 °C	*
	R: TTACCGCGGCTGCTGG			
*Bf*	F: CCTGACTAAGAAGCACCGGC	107 bp	60 °C	U41167.1
	R: GTAAAACCGCCTACGCTCCC			
*Rf*	F: TATCTTAGTGGCGGACGGGT	157 bp	60 °C	MT356193.1
	R: TCTAATCAGACGCGAGCCCA			
*Fi*	F: AACTCCACGTGTGGGATGAA	164 bp	60 °C	NR_042150.1
	R: CCAGTGATTCCGAACAACGC			
*Pr*	F: GGATGGGGATGCGTCTGATT	129 bp	60 °C	NR_028866.1
	R: CTGCCTCCCGTAGGAGTTTG			
*Su*	F: CGTCCGATTAGCTGGTTGGT	185 bp	60 °C	NR_026205.1
	R: AAGAACTTCCTCACCCACGC			
*Cb*	F: GCAACGCGAAGAACCTTACC	110 bp	60 °C	NR-042144.1
	R: GCGGGACTTAACCCAACATC			
*Sr*	F: GAATCATTGGGCGTAAAGGG	140 bp	60 °C	AB198442.1
	R: CATTTCACCGCTACACTAGG			
*Tb*	F: CCTTATGTCCAGGGCTACAC	115 bp	60 °C	NR-118718.2
	R: CGGGTTTCAGACTCCTATCC			

* indicated that the bacterium was the steward gene sequence (the 16S rRNA sequence).

## Data Availability

The authors affirm that all data necessary for confirming the conclusions of the article are present within the article, figures, and tables.

## Supplementary Materials

The following supporting information can be downloaded at https://www.mdpi.com/article/10.3390/ani14050788/s1. Data S1: Microbial composition. Data S2: Microbial differential metabolites (positive ion mode). Data S3: Microbial differential metabolites (negative ion mode). Figure S1: Differential metabolite–differential microbiota correlation heat map.
