# Supplementary material for: Supplementation with Astragalus Root Powder Promotes Rumen Microbiota Density and Metabolome Interactions in Lambs
Source: Animals (Basel). 2024 Mar 2;14(5):788. doi: 10.3390/ani14050788 (PMC10931105; doi:10.3390/ani14050788)
Supplement: Supplementary file 1 [file animals-14-00788-s001.zip › Figure S1.pdf]

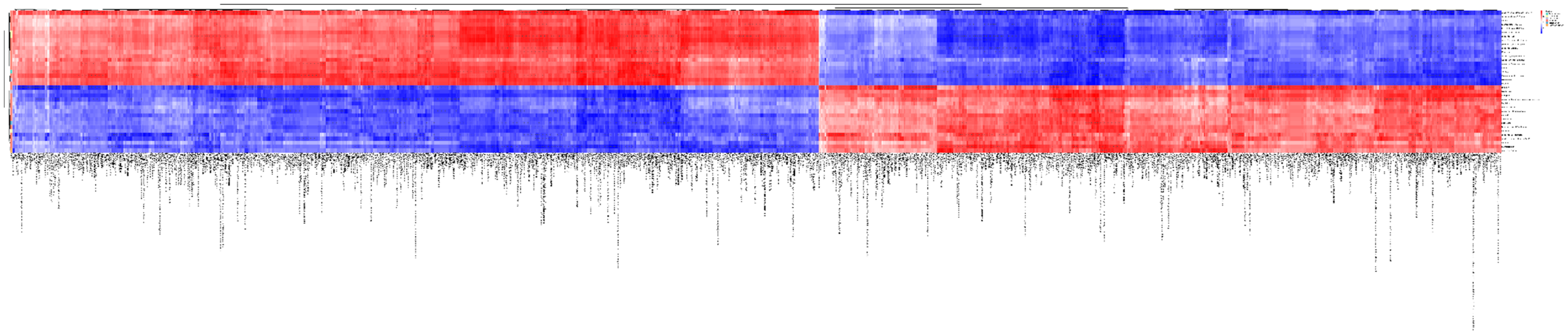

Figure S1 Differential metabolite-differential microbiota correlation heat map

Note: The right side of the figure shows the classification of differential microbiota (genus level), the bottom shows differential metabolites, and the left and top show the clustering dendrogram of differential microbiota and differential metabolites, respectively. Different colors represent the magnitude of Spearman's correlation coefficient; the closer the absolute value is to 1, the higher the correlation. Red color indicates positive correlation, while blue color indicates negative correlation, and the darker the color, the stronger the correlation. Asterisks indicate significant correlation ($P<0.05$) between differential metabolites and differential microbiota, and *, ** and *** indicate that the degree of significance of the correlation is increasing in the order of $P<0.05$, $P<0.01$ and $P<0.001$, respectively.
